# AhR-deficiency as a cause of demyelinating disease and inflammation

**DOI:** 10.1038/s41598-017-09621-3

**Published:** 2017-08-29

**Authors:** Ludmila Juricek, Julie Carcaud, Alice Pelhaitre, Thorfinn T. Riday, Aline Chevallier, Justine Lanzini, Nicolas Auzeil, Olivier Laprévote, Florent Dumont, Sebastien Jacques, Frank Letourneur, Charbel Massaad, Cendra Agulhon, Robert Barouki, Mathieu Beraneck, Xavier Coumoul

**Affiliations:** 1 INSERM UMR-S1124, Toxicologie Pharmacologie et Signalisation cellulaire, Paris, France; 2 CNRS UMR8119, Centre de Neurophysique, Physiologie, Pathologie, Paris, France; 3 CNRS UMR8638 Chimie Organique, Médicinale et Extractive et Toxicologie Expérimentale, Paris, France; 4 Plate-Forme Séquençage et Génomique, Institut Cochin, Paris, France; 5 CNRS FR 3636, Glia-Glia and Glia-Neuron Interactions Group, Paris, France; 6 Université Paris Descartes, 45 rue des Saints-Pères, 75006 Paris, France

## Abstract

The Aryl hydrocarbon Receptor(AhR) is among the most important receptors which bind pollutants; however it also regulates signaling pathways independently of such exposure. We previously demonstrated that AhR is expressed during development of the central nervous system(CNS) and that its deletion leads to the occurrence of a congenital nystagmus. Objectives of the present study are to decipher the origin of these deficits, and to identify the role of the AhR in the development of the CNS. We show that the AhR-knockout phenotype develops during early infancy together with deficits in visual-information-processing which are associated with an altered optic nerve myelin sheath, which exhibits modifications in its lipid composition and in the expression of myelin-associated-glycoprotein(MAG), a cell adhesion molecule involved in myelin-maintenance and glia-axon interaction. In addition, we show that the expression of pro-inflammatory cytokines is increased in the impaired optic nerve and confirm that inflammation is causally related with an AhR-dependent decreased expression of MAG. Overall, our findings demonstrate the role of the AhR as a physiological regulator of myelination and inflammatory processes in the developing CNS. It identifies a mechanism by which environmental pollutants might influence CNS myelination and suggest AhR as a relevant drug target for demyelinating diseases.

## Introduction

The Aryl hydrocarbon Receptor (AhR), a ligand-activated transcription factor, belongs to the basic-Helix-Loop-Helix/Per-ARNT-Sim family that regulates detoxification^1^. Recent discoveries using knockout models have suggested that the AhR has distinct roles in mammalian physiology^2, 3^. The modulation of AhR expression causes defects during neuro-development in model organisms such as C. *elegans*. Although the AhR mRNA is expressed in various cerebral regions^4–8^, its role in the development and functions of the nervous system in vertebrates remains elusive. Previously, we have shown that the AhR-knockout (AhR-KO) adult mice display a spontaneous horizontal nystagmus of unknown origin^9^. In humans, the diagnosis and the characterization of the nystagmus have major clinical implications because it represents a predictive symptom of major central nervous system pathologies, including multiple sclerosis or Pelizaeus-Merzbacher disease^10–13^. Nystagmus is also encountered in specific neuro-developmental diseases grouped under the term of Infantile Nystagmus Syndrome (INS). The mechanisms involved in the establishment of such abnormal eye movements during development in infants have not yet been characterized because of their heterogeneity and of the lack of animal models^14^. We have previously shown that the origin of the nystagmus was not vestibular neither cerebellar, while the AhR was expressed in the eye during mice development. We have now extended our research at the cellular and molecular levels focusing our efforts on different characteristics of the visual circuitry, mostly anatomical and structural, and showed that the disease was in fact related to a significant demyelination of the optic nerves in the AhR-KO mice. A multi-omics non-candidate approach then revealed that the mechanism of this demyelination is related to an inflammatory state of the optic nerve which impairs the expression of the myelin-associated glycoprotein (MAG) as demonstrated by *in vivo* and *ex vivo* approaches.

Overall, our study first reports the role of the AhR in the pathogenesis of demyelinating diseases associated with inflammation such as optic neuritis or multiple sclerosis, providing new avenues for the understanding and the treatment of nystagmus-related pathologies.

## Results

### Functional characterization of gaze–related pathways

**Figure 1:**
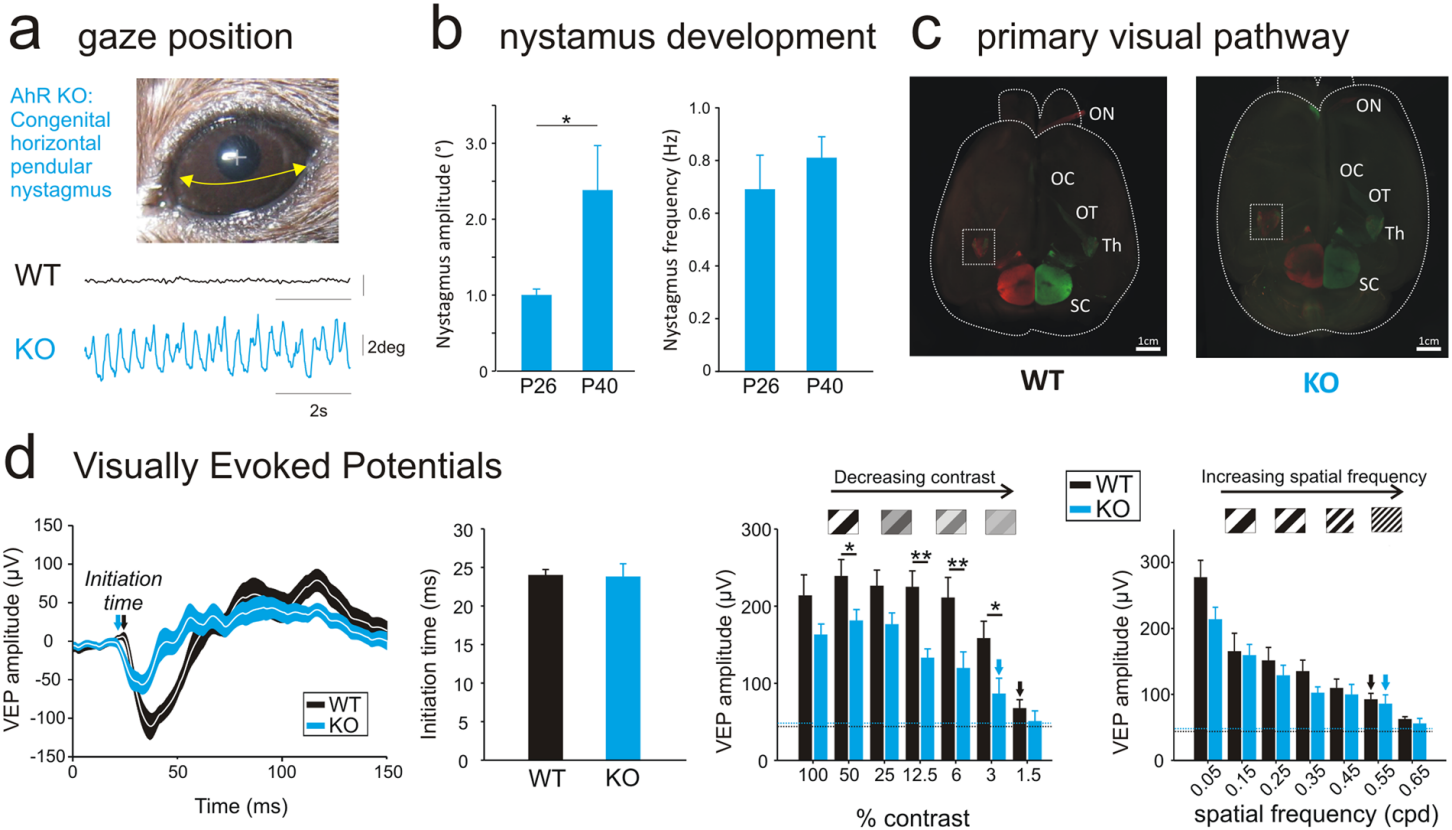
AhR knockout mice as a model of INS pathologies. (**A**) Raw traces of spontaneous eye movements recorded in the presence of light (8 week-old AhR-KO). (**B**) Nystagmus amplitude (degrees) and frequency (Hz) recorded at P26 and P40 (n = 8). (**C**) Example of whole brain staining of a WT and an AhR KO mouse: Optic Nerve (ON), Optic Chiasm (OC), Optic Tract (OT), Thalamus (Th) and Superior Colliculus (SC). (**D**) Left: Average Visually Evoked Potentials (VEP) traces recorded in WT (black line, n = 9) or AhR KO mice (blue line, n = 8) (100% contrast and 0.05 cpd stimulation). The white line represents the average and the envelope around (blue or black) represents the SEM. Middle: Initiation time of the VEPs measured on both WT (black bar) and AhR KO mice (blue bar). Right: VEP amplitudes with decreasing contrast stimulation or decreasing visual acuity (i.e. increasing spatial frequency). Dotted lines represent the noise. Arrows represent the first stimulation significantly different from the noise. (*p < 0.05; **p < 0.01).

AhR-KO mice exhibit a spontaneous pendular horizontal nystagmus (Fig. 1a), increased by the presence of visual inputs (Figure S1a)^9^. To determine whether it is acquired during postnatal development, video-oculography was performed on young mice (P26–40) (Figs 1b and S1b). At P26, the nystagmus was present in all AhR-KO mice, while it was never observed in WT-control mice. During the period of functional maturation of gaze stabilizing reflexes^15^, there was a significant increase of the amplitude of the oscillations (Figure S1b). These data demonstrate that AhR-KO congenital nystagmus appears early in development and worsens with aging. Next, we tested the functionality of the optomotor pathways (Figure S1c). While all available models have gross abnormalities in the optokinetic reflex^11, 16^, it was reduced but present and in compensatory direction in all tested AhR-KO mice (Figure S1c). Then, we checked whether the nystagmus could originate from anatomical defects as previous INS-models were shown to be achiasmatic^17, 18^. Examinations of the projections of the optic nerves to the thalamus demonstrated no anatomical differences between WT and AhR-KO mice (Figs 1c and S1d). Considering that nystagmus is often associated with visual deficits, we recorded visual evoked potentials (VEPs) from the primary visual cortex; we found that amplitudes were lower in adult AhR-KO vs. WT mice (Fig. 1d-left), indicating a deficit in the integration of visual information. While we did not observe a significant difference in the initiation time of the VEPs between AhR-KO and WT groups, indicating that velocity conduction is preserved in AhR-KO mice (Fig. 1d-middle), the presentation of different visual stimuli demonstrated a ≈32% decrease in the sensitivity to contrast, but not in the visual acuity (Fig. 1d-right). This sensitivity appears to be critical for the mice to discriminate orientations^19^. These results demonstrate that the AhR-KO nystagmus is associated with a functional deficit in the early processing of visual information.

### AhR KO mice display structural alterations of the myelin of optic nerves

**Figure 2:**
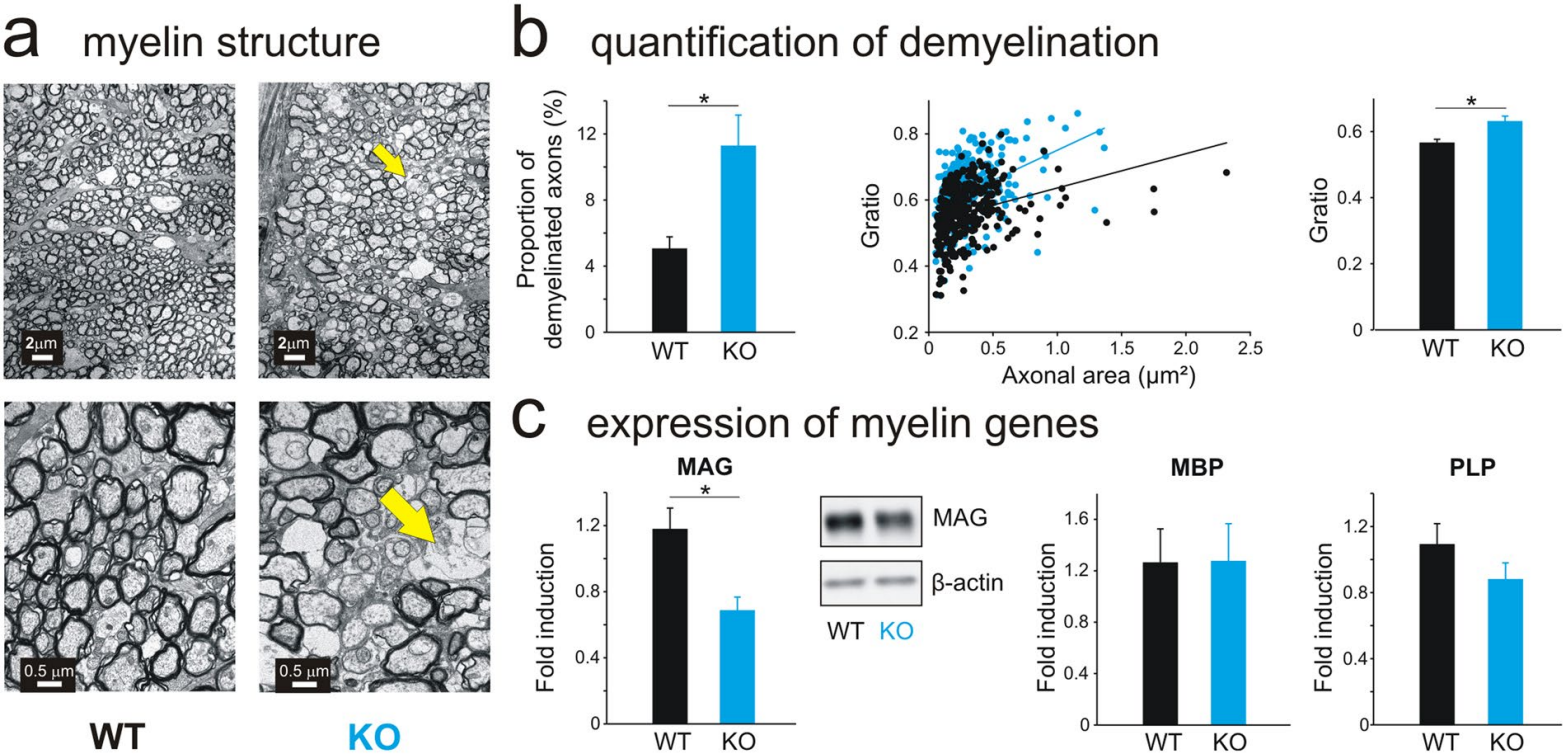
AhR deficiency leads to myelin alterations in the optic nerve. (**A**) Example of transverse ultrathin sections of the optic nerve of 8 weeks-old WT (left) and AhR KO (right) mice examined by transmission electron microscopy. Scale bars: 2 µm (top) or 0,5 µm (bottom). The yellow arrows indicate zones of disorganized myelin sheath. (**B**) **Left**: percentage of low-myelinated axons in the optic nerves of WT (n = 5, black bar) and AhR-KO (n = 5, blue bar) mice. Middle: Scatter plots depicting G-ratio (ratio of axon diameter/myelinated fiber diameter) of WT (n = 352 axons, black) and AhR KO (n = 243 axons, blue) mice. Right: Quantification of the G-ratio of optic nerves for each genotype (n = 5 WT and n = 5 AhR KO). (**C**) Quantitative Real-Time PCR analysis of three myelin genes (MAG, MBP, PLP) in the WT (black bars) and AhR-KO (blue bars) mice optic nerves (n = 18–19/group). mRNA levels were normalized to GAPDH mRNA expression. Middle: MAG protein in whole tissue lysates of WT and AhR-KO mice optic nerves was detected by Western Blot. Total actin was used as a loading control (n = 4/group). A full-length gel is included in the supplementary file (complete western blot). MAG: myelin associated-glycoprotein; MBP: myelin basic protein; PLP: proteolipid protein; GAPDH: glyceraldehyde 3-phosphate dehydrogenase (*p < 0.05).

Concomitantly, as nystagmus occurs frequently in demyelinating pathologies^12, 13, 20^, we hypothesized that AhR-KO mice could present structural alterations of the myelin sheath. Electron-microscopy analyses of the optic nerves revealed a disorganization of the myelin sheath of AhR-KO mice (Fig. 2a). Furthermore, the proportion of low-myelinated axons and the G-ratio, a structural index of optimal axonal myelination (Fig. 2b), were significantly increased in AhR-KO mice. As the myelin sheath is mainly composed of complex lipids and proteins, we then investigated its composition and performed a lipidomic analysis to determine if the structural defects were due to a modification in the lipid content. Subsequent univariate and multivariate analyses showed significant decreases for 28 lipids in AhR-KO *vs*. WT mice, especially the ceramides family (Table S1) which is specific of the central nervous system. Afterwards, we assayed the mRNA and protein expression of genes which play a crucial role in the formation of the myelin sheath in the optic nerve^21^: qRT-PCR analysis revealed a down-regulation of MAG mRNA levels in adult AhR-KO mice *vs*. WT mice, which was confirmed at the protein level (Fig. 2c, left). This effect is specific to MAG as the levels of myelin basic protein (MBP) and proteolipid-protein (PLP) mRNA levels remained unchanged between AhR-KO and WT mice (Fig. 2c, right). Altogether these results suggest that the alteration of the myelin sheath surrounding the neurons of the optic nerve could originate in the altered lipid content combined with the down-regulation of MAG.

### Pro-inflammatory cytokines are increased in the optic nerves of AhR KO mice

**Figure 3:**
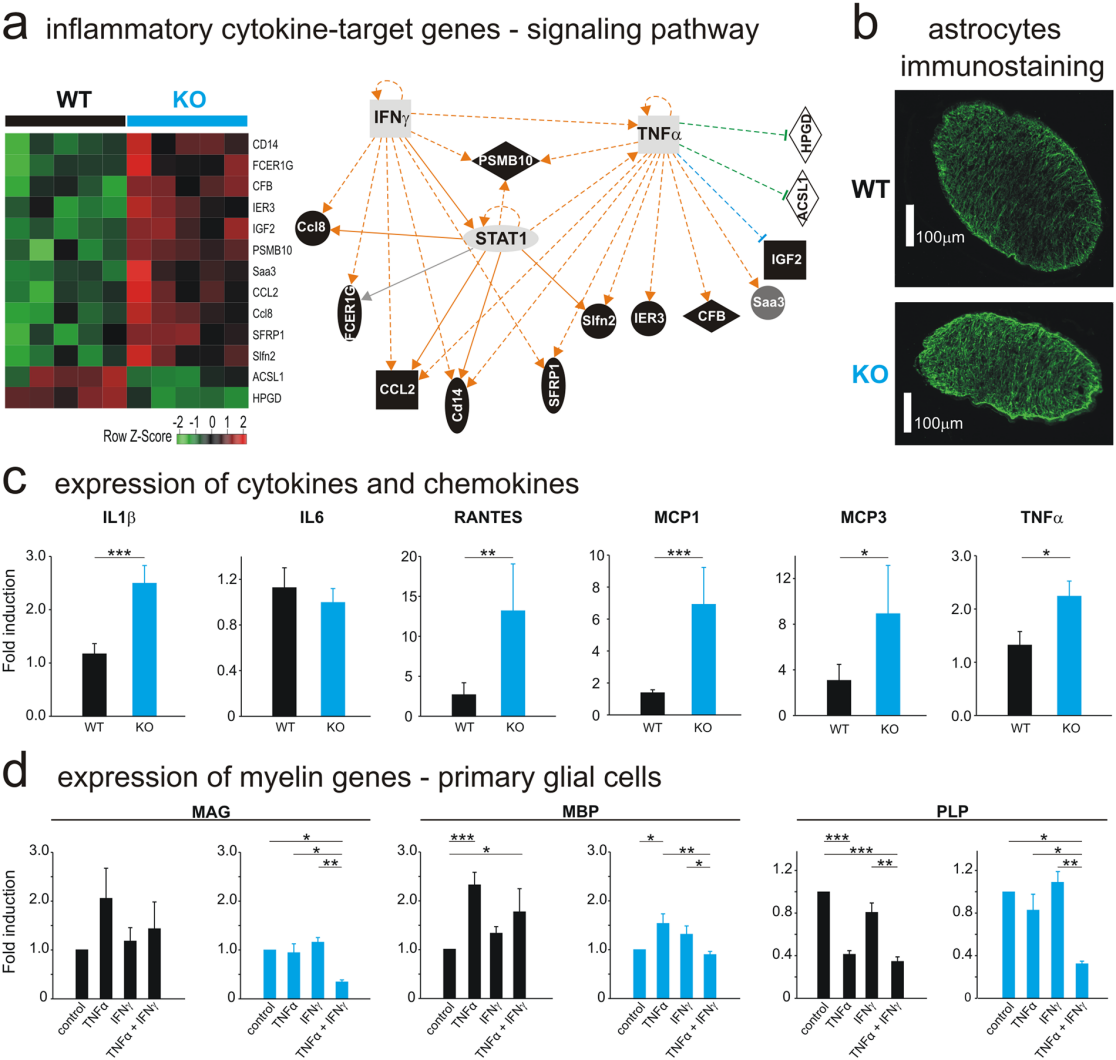
AhR-dependent inflammatory response controls myelin genes. (**A**) Left: Heatmap of the annotated genes with the highest importance in inflammatory response. Red: increased mRNAs expression; green: decreased mRNA expression (n = 5/group). Right: Connected components of the relevance network after IPA. Thirteen gene probes were analyzed by IPA software and annotated for gene network construction. Both over-expressed and under-expressed genes were mapped to the same network space. The pathway analysis revealed a central regulating role for tumor necrosis factor-alpha (TNF-α) and interferon-gamma (IFNγ). Main components of the legend: hatched line: indirect interaction; continuous line: direct interaction; arrow: acts on; line with a perpendicular line at the end: inhibits; square: cytokine (Interferon-γ and TNF-α); horizontal oval (STAT1); transcription regulator; vertical oval (SFRP1): transmembrane receptor; vertical diamond (ASCL1): enzyme; horizontal diamond (CFB): peptidase; circle (Saa3): other. (**B**) Expression of immunoreactive GFAP, an astrocyte marker, in the optic nerve of WT (top) and AhR KO (bottom) adult mice (Scale bar: 100 µm, n = 4/group). **(C)** Quantitative Real-Time PCR analysis of Interleukine-1β (IL1β), IL6, RANTES (Regulated on Activated Normal T-cell Expressed and Secreted), MCP1–3 (Monocyte Chemoattractant Protein 1–3), Tumor Necrosis Factor-α (TNF-α) in the WT (black bars) and AhR-KO (blue bars) mice optic nerves (n = 10–22/group). mRNA levels were normalized to GAPDH mRNA expression. (**D**) Myelin genes (MAG, MBP and PLP) mRNA expression was determined by Quantitative Real-Time PCR analysis in primary mixed glial cell cultures after 3 days-treatment with TNFα (10 ng/mL), IFNγ (10 ng/mL) or both (concentration 10ng/mL of each) or vehicle (control). The relative mRNA expression levels were represented as relative fold change compared to mRNA abundance in cells treated with vehicle. Data are expressed as the mean of four to six independent experiments. Each experiment consists of pool of 2–8 pups. IPA: Ingenuity Pathway Analysis, GFAP: Glial Fibrillary Acidic Protein (***p < 0.001, **p < 0.01; *p < 0.05).

In order to identify the signaling pathways involved in all observed phenomenon at the functional, cellular and molecular levels (nystagmus, demyelination, MAG expression), we investigated the transcriptomes of the AhR-KO and WT optic nerves. Heat map analysis revealed that 2366 genes were differentially (p < 0.05) expressed in AhR-KO mice (Figure S2a). Ingenuity Pathway Analysis identified inflammatory pathways among the top scoring interaction networks (Figure S2a). Hierarchical clustering suggested a central regulating role of Signal Transducers and Activators of Transcription 1 (STAT1) protein (Fig. 3a) which is activated for example by Tumor Necrosis Factor-α (TNFα) as demonstrated by the direct association of STAT1 with TNFα Receptor 1^22^. Inflammation in the optic nerves of AhR-KO mice was confirmed by immunostaining showing an increase of the expression of the glial fibrillary acidic protein (GFAP) in astrocytes (Fig. 3b). In contrast, we did not observe a significant increase of the expression of Iba1 in microglia (Figure S2b). Moreover, the expression of pro-inflammatory cytokines and chemokines (Interleukine-1β, Regulated on Activated-Normal T-cell Expressed and Secreted; Monocyte Chemoattractant Protein 1–3; Tumor Necrosis Factor-α) genes significantly increased (Fig. 3c). To determine how the inflammatory environment affects the expression of myelin genes, we examined the effects of TNFα and/or interferon-gamma (IFN-γ) which activates the STAT1 pathway^22^, alone or in combination, in mixed-glial cell cultures (which contain oligodendrocytes, expressing MAG and responsible for myelination) after 3 days of treatment (Fig. 3d). Interestingly, these two pro-inflammatory cytokines taken individually have no effects on MAG expression, whereas the co-treatment significantly decreases MAG mRNA expression by approximately 60% only in AhR-KO mice, indicating that this down-regulation is AhR-dependent (Fig. 3d-left). Moreover, the co-treatment further increased the mRNA expression of most cytokines and chemokines studied, which demonstrates the existence of a positive feedback loop (Figure S2c) susceptible to further aggravate inflammation. Collectively, these data suggest a causal link between the expression of the AhR, the inflammatory process found in the optic nerves of AhR-KO mice, the reduction of MAG expression, and the myelin disruption.

## Discussion

Our study has identified the AhR and its role in the myelination of the visual and optomotor pathways as a putative cause for Infantile Nystagmus Syndrome (INS). Hence, the AhR-KO is a developmental model which shares numerous features with the human pathology^14^. First, AhR KO mice display as early as on P26 a nystagmus that rapidly aggravates with age. Because of the initial low amplitude and low frequency of the eye movements, it would be difficult to detect any deficit beforehand; however, this result suggests that, as in infants, the nystagmus appears during early maturation of the visual system. Other characteristics of the AhR-KO phenotype resemble the INS: abnormal eye movements are essentially horizontal, aggravate with light or fixation attempts, and evolve with age. Finally, while previous animal models of INS were achiasmatic and had inverted optokinetic responses^11, 16–18^, we show that the AhR-KO mice has normal decussating retinal projections and show compensatory responses during optokinetic stimulation, both features observed in INS^10^. Interestingly, this study highlights, for the first time, that the AhR is involved in myelination of central nervous system. Fernandez *et al*. have shown that a single prenatal exposure to TCDD, an exogenous AhR ligand, could disturb the expression of oligodendrocyte markers during the rat brain development but did not investigate on the myelin structure^23^. In our model, the deletion of AhR provokes a severe disorganization of myelin sheath surrounding axons of retinal ganglion cells, indicating that this receptor plays an important role in myelin structure and maintenance. The strong and significant decrease of galactosylceramide (GalCer) concentrations measured by UPLC-MS in the optic nerve of AhR-KO mice suggest that modified ceramides might represent potential biomarkers of inflammatory diseases in the CNS as also suggested by Mayo *et al*. who characterized increased levels of lactosylceramide (LacCer) during chronic experimental autoimmune encephalomyelitis (EAE), a model of multiple sclerosis (MS)^24^. One of the first indicators of MS in humans is a nystagmus. LacCer which is produced by β-1,4-galactosyltransferase 6 (whose levels increase in EAE) controls astrocyte and microglia activation and then promotes neurodegeneration^24^. Taken together these data demonstrate that an activation or a deletion of this receptor may interfere with physiological myelination process.

In addition to its alterations of the myelin structure of the optic nerves, AhR-KO mice also develop a local inflammatory environment. Indeed, microarrays studies have shown that AhR-KO mice display an inflammatory optic nerve resulting in the secretion of pro-inflammatory cytokines, which likely amplify the local inflammation. This is in line with other studies showing that the AhR-KO mice are inflammation prone. It was shown for instance, that these mice develop a severe form of experimental autoimmune encephalomyelitis^25^. Moreover, the AhR inhibits the transcription of NLRP3 which is important for the inflammasome activity^26^. In line with our study, TNF-alpha production is also significantly higher in AhR-deficient macrophages stimulated by the lipopolysacharride (LPS)^27^. Interestingly, an inflammation of the optic nerve, or optic neuritis, is often associated in humans with acquired demyelinating pathologies such as multiple sclerosis^26^ or leukodystrophies^12^, in which the nystagmus serves as a symptomatic biomarker and usually signs an inflammatory state^14^.

Recent studies have shown that myelin-related diseases can be associated with a 30% up- or down-regulation of myelin genes^28, 29^. This is also in line with our results, as we observed a >40% decrease of MAG expression. Moreover, MAG deficiency has been associated with the occurrence of a nystagmus in myelin-associated diseases^29^. MAG is expressed by oligodendrocytes and regulates the myelin attachment to the neuron together with a signaling function. Because in our model the AhR was knocked-out in all neural cells, whether the myelin impairment relates to the AhR-deficit specifically in the oligodendrocytes remains to be determined. Here, we demonstrated for the first time, that the inflammatory environment, as the combination of TNF-alpha and IFN-gamma, is key in an AhR-deficient context to decrease the expression of MAG; interestingly, we also observe a strong modification of PLP mRNA expression in primary mixed glial cell cultures from both origins (WT and KO). In WT primary mixed glial cell cultures, the effect is primarily due to TNF-alpha as IFN-gamma did not impact the mRNA expression of PLP nor modify the effect of TNF-alpha. In KO primary mixed glial cell cultures, the effect is on the other hand primarily due to the mixture TNF-alpha and IFN-gamma. The significant effect of the cytokine mixture on MAG mRNA expression specifically in KO primary mixed glial cell cultures suggests that the KO phenotype sensitize the oligodendrocyte to the combined effects of both cytokines; this also strengthens our experiments on KO optic nerves and provides a mechanistic link explaining this decrease of expression: we can indeed hypothesize that the absence of the AhR leads to an inflammatory state characterized by an increase secretion of TNF-alpha and IFN-gamma which decrease the expression of MAG. Several hypotheses could explain the differential effects on PLP expression observed when comparing optic nerves and primary glial cells: (1) the dose of cytokines used as a treatment on the primary glial cells might have been higher than *in vivo*, and would be sufficient to promote a decreased expression of PLP compared to the concentrations in the optic nerve, which only acts on MAG expression. (2) the primary mixed glial cell culture is a model which is not fully representative of the *in vivo* situation as e.g. the lack of neurons, which could sensitize differentially the oligodendrocyte regarding PLP expression. These results nevertheless establish a causal link between the AHR, the occurrence of an inflammatory state, and the regulation of a myelin-associated proteins and that this potential link should be studied in the future, focusing on inflammatory-related pathologies of the central nervous system.

## Conclusions

Overall, the AhR-KO model clearly exhibit many cellular and functional features associated with inflammatory demyelinating diseases. Our observations indicate that the AhR represents a relevant pharmacological target in order to modulate inflammatory and demyelinating processes, providing new avenues for the understanding and the treatment of these pathologies in humans.

## Methods

### Mice

The European Communities Council Directive 2010/63/EU on the protection of animals was followed for the experiments using animals. All procedures were approved by the ethical committee for animal research of the University Paris Descartes (CEEA.34). AhR KO mice are a generous gift of Pr. PM Fernandez-Salguero^30^.

### Video-oculography

#### Animals and surgery

Eye-movement recordings were performed on a total of 32 mice, including 26 AhR KO and 6 AhR WT. Surgical preparation and postoperative care for head implant surgery have been described previously^9^. Briefly, Gas anesthesia was induced using isoflurane and a small custom-built head holder was cemented (C&B Superbond) to the skull just anterior to the lambda landmark. Following the surgery, animals were isolated and closely surveyed for 48 h. Buprenorphine (0.05 mg/kg) was provided for postoperative analgesia and care was taken to avoid hypothermia and dehydration. Surgery was performed on wild-type mice and AhR KO mice, either between postnatal day 21 and 24 (P21–24) for the experiment on the onset of nystagmus, or at postnatal day 56 (P56) for the characterization of adult nystagmus and optokinetic deficit tests.

#### Data acquisition

The experimental set-up and methods of data acquisition and analysis used to record eye movements were similar to those previously described^9^. Briefly, Mice were head-fixed at a ~30° nose-down position to align the horizontal canals in the yaw plane. Animals were placed in a custom built Plexiglas tube, and eye movements were recorded using an infrared video system (ETL-200, ISCAN, Burlington MA). Eye position signals were sampled at 1 kHz, digitally recorded (CED power1401 MkII) with the Spike 2 software and later exported into the Matlab (The MathWorks) programming environment for off-line analysis.

#### Data analysis

Horizontal and vertical eye movement data were digitally low pass-filtered (cut-off frequency: 40 Hz), and position data were differentiated to obtain velocity traces. Segments of data with saccades were excluded from analysis. The frequency and the amplitude of the nystagmus were measured using custom-made software written in Matlab (Mathworks). Because AhR KO mice show spontaneous nystagmus, OKR responses were measured as the mean eye velocities on segments longer than 2/Fn where Fn is the measured frequency of the spontaneous nystagmus. OKR gains were then calculated as the ratios of the mean eye velocities to the constant visual stimulus velocity.

#### Protocol for eye movement recordings

Mice were securely head-fixed in the apparatus and isolated from the experimental room under a 40 cm wide dome. All sources of light were turned off except for the optokinetic projector. Light intensity inside the dome was measured at 185 Lux during light conditions and <0.02 Lux during dark conditions (Luxmeter Lux-1337 Iso-tech). The different tests were performed as follows: i) animals were left unperturbed during an initial accommodation period of 5 minutes; ii) spontaneous eye movements were recorded in the light and then under dark conditions. Recordings typically lasted between 3–6 min for each condition; iii) in adults, visuo-motor responses were tested further during optokinetic stimulation. Optokinetic full field stimulation was performed by projecting a dotted pattern at velocities in a range of 2.5–7.5°/s in alternated clockwise and counter-clockwise directions (S. Fig. 1c). The dotted pattern consisted of 25000 white dots (max width 0.075°) randomly distributed on a black background. Visual stimulations lasted 1 min and were separated by 2 min in the dark. Analysis was performed as previously reported^9^.

### Anatomical staining using CTB

Optic nerves fiber projections were visualized using an anatomical staining and ultramicroscopy method in 5 WT mice and 7 AhR KO mice aged 9 to 29 weeks^31^. The animals were anaesthetized using a mixture of ketamine (80 mg/kg) and xylazine (15 mg/kg) injected intra-peritoneally. Alexa 488 or Alexa 594 conjugated to cholera toxin subunit B (CTB; Life Technologies, France) were diluted in PBS (2 µg/mL), and 2 µL of either solution was injected into the vitreous humor of the right or left eye, respectively. After 3 days, the mice were deeply anesthetized, perfused and the brains removed and post-fixed for a 3–4 hours in PFA 4% in PBS. Then, the brains were made transparent using an adapted version of the 3DISCO method^32^. They were incubated in the following solution at room temperature under agitation: overnight in 50% tetrahydrofuran (THF, Sigma-Aldrich, France), 2 h in 80% THF, two times 1 h in 100% THF, 45 min in dichloromethane (DCM, Sigma-Aldrich, France) and finally in dibenzylether (DBE, Sigma-Aldrich, France) for storage until image acquisition. Ultramicroscopy was then performed using a 100% quartz imaging reservoir where the light sheet illuminates and crosses cleared samples^31^. Sheet serial imaging at 488 (power 85%) and 561 nm (power 90%) wavelengths were performed (Coherent Sapphire Laser, LaVisionBiotec) in 2 µm incremental steps of the entire thalamus using the 4x objective. High-resolution 16 bit images were then processed using Imaris 7.6.1 software, and 3D reconstructions of the thalamus were obtained. Only cases with complete retinal fills, showing good staining of the thalamus, were used for the quantification of the retinal projections. The volumes of the ipsilateral and contralateral staining, i.e. the volumes of stained voxels, were then calculated and the ratio R = Vipsi/Vcontra was calculated to assess nerve fiber projections.

### VEP recordings

Mice were anaesthetized with intraperitoneal injections of ketamine (120 mg/kg) and xylazine (9 mg/kg) for surgery, and a local anesthetic (1% lidocaine) was applied. Tungsten microelectrodes (300–500 kΩ, FHC) were bilaterally implanted into the binocular zone of the visual cortex (3 mm lateral of lambda) at a depth of 450 µm below the cortical surface corresponding to layer 4; reference electrodes were inserted at the brain surface, 2 mm lateral and 1 mm posterior to bregma. Electrodes were secured in place using cyanoacrylate, and a post was fixed to the skull anterior to the bregma for head fixation. Recordings were performed 48 h after the surgery for recovery; all mice were first habituated to the experimental set-up 24 h before visual evoked potential (VEPs) recordings. Stimuli consisting of full-field sine wave gratings of 100% contrast with different acuity (0, 0.05, 0.15, 0.25, 0.35, 0.45, 0.55 or 0.65 cycles per degree or cpd) or of 0.05 cpd with different contrast (100, 50, 25, 12.5, 6, 3 or 1.5%) were presented at 20 cm in front of the animal on a 17-inch cathode ray tube monitor. Visual acuity and contrast sensitivity were determined by the trough-to-peak VEP amplitude of the 120 averaged responses per condition (8 blocks, 15 presentations in random order). Visual thresholds, corresponding to noise level, are represented as dashed lines (Figs 1e and 3c right) and were calculated using the VEP amplitude during the presentation of a grey screen. For all mice, the data from the bilateral electrodes were pooled. All recordings were amplified, band pass filtered (between 0.4 and 100 Hz) and acquired with a Micro3-1401 digitizer (CED Cambridge). Off-line analysis was performed under spike2 environment.

### Transmission electron microscopy

Ultrathin sections (50–90 nm) of optic nerves were cut on an ultramicrotome (8800 Ultrotome III; LKB Bromma) and collected on 300-mesh nickel grids. Staining was performed with 4% (vol/vol) aqueous uranyl acetate, followed by Reynolds lead citrate. Ultrastructural analyses were performed in a JEOL JEM-1011 electron microscope and digitalized with DigitalMicrograph software. Image acquisition was performed at the Cochin Institute imaging facility. The G-ratio (ratio of axon diameter/myelin + axon diameter) was determined with ImageJ software (National Institutes of Health). At least 300 randomly selected axons were analyzed per animal (5 WT mice and 5 AhR KO mice). Healthy axons were defined on the basis of the presence of intact membranes and the normal complement of organelles.

### Transcriptome and qRT-PCR analysis

#### RNA sampling from optic nerves (including tissue extraction, RNA purification, checking of RNA purity)

Optic nerves were placed in 1 mL of Qiazol® reagent with 2 stainless steel beads (Qiagen, Courtaboeuf, France) and were homogenized with a Tissuelyser system (RetschMM300, Germany). Total RNA was prepared using the RNeasy Mini Kit following the manufacturer’s instructions (Qiagen, Les Ulis, France). The quality of total RNA was monitored with a Nanodrop ND-1000 spectrophotometer (Nanodrop products, Wilmington, DE, USA).

#### Quantitative real-time PCR

Reverse transcription was performed using the High Capacity cDNA Reverse Transcription Kit (Life technology, France) according to the manufacturer’s directions. Then quantitative real time polymerase chain reaction was performed with 20 ng of cDNA, with duplicates for each experiment. Table S3 gives the gene-specific primers. The relative amounts of mRNA were estimated using the ΔΔC_T_ method with glyceraldehyde 3-phosphate dehydrogenase (GAPDH) as the reference gene^33^.

#### Transcriptome analysis (including hybridization and analysis)

Transcriptome analysis was performed with Affymetrix Mouse Gene 1.0 ST arrays (a genome wide array with 28000 probe sets) according to the manufacturer’s protocol. Briefly, total RNA (300 ng) was labeled and cRNA (antisens RNA) was synthetized using the Affymetrix WT cDNA Synthesis and Amplification Kit. After cleanup protocol, ssDNA (sense single stranded DNA) was synthetized, fragmented and labeled with biotin. Biotin-ssDNA was hybridized onto microarrays according to the manufacturer’s instructions. After 16 h at 45 °C, microarrays were washed and stained using Affymetrix fluidics station 450 and scanned with an AffymetrixGeneArray scanner 3000 7G. Raw data were normalized using the Robust Multichip Algorithm (RMA) in Bioconductor R^34, 35^. Then all quality controls and statistics were performed using Partek GS® (version 6.6 Copyright© 2012 Partek Inc., St. Louis, MO, USA). Hierarchical clustering (Pearson’s dissimilarity and average linkage) and principal component analysis comprised unsupervised exploratory data analyses. These multivariate technics aim to assess data for experimental bias or outlier samples. To find differentially expressed genes, classical analysis of variance (ANOVA) was performed for each gene and pair wise Tukey’s post hoc tests between groups. p-values and fold changes were used to filter and select differentially expressed genes.

#### Functional analysis and data submission

Interaction, pathway and functional enrichment analyses were carried out with IPA (Ingenuity® Systems, www.ingenuity.com, USA). All data obtained by microarray analysis have been submitted on GEO Omnibus site.

### Immunobloting

Optic nerves were collected into RIPA buffer (PBS, 0,5% deoxycholate, 0,1% SDS, 1% NP40) containing protease (1%) and phosphatase (1%) inhibitors (Sigma), in presence of 2 stainless steel beads in order to be homogenized with TissueLyser system. Three micrograms of proteins were separated by SDS-PAGE and transferred onto a nitrocellulose membrane. The membrane was blocked in 5% skimmed milk during 2 hours, followed by overnight incubation at 4 °C with anti-MAG antibodies or β-actin (Abcam, France). After three washes with TBS-0,1%Tween20, the membrane was incubated with the corresponding HRP-labeled secondary antibodies (Cell Signaling). Then, the membrane was observed using ImageQuant LAS 4000 (GE Healthcare).

### Immunofluorescence staining

After sacrifice, optic nerves were removed, postfixed overnight in 4% paraformaldehyde at 4 °C, then cryoprotected overnight in 10% sucrose at 4 °C, and subsequently frozen in gelatin-sucrose. Optic nerves (10 µm cryostat slices) were permeabilized for one hour with 0,3% Triton-PBS. Incubation with GFAP and Iba1 antibodies (Abcam) diluted in 0,3% Triton-PBS was performed overnight. After three washes with PBS, optic nerves were incubated with the corresponding Alexa-488-labeled chicken antibodies (Invitrogen). The coverslips were sealed with Dako Paramount Aqueous Mounting Medium Ready-to-use (Invitrogen). Tissues were observed and images recorded using an Epifluo Nikon Eclipse TE-2000E microscope with Plan fluor 10x/0,30,DIC L/N1, O.N = 0,30, D.T = 16 mm.

### *Ex vivo* culture and treatment

Primary glial cells (oligodendrocytes and astrocytes) were prepared from P2–3 mouse brain from WT or AhR KO mice. Briefly, meninges were removed and cerebral hemispheres were mechanically dissociated in medium. Cell suspensions were plated in six-well plates coated with 20 µg/mL poly-D-lysine (Sigma-Aldrich). Cells were then incubated during 21 days *in vitro* (DIV21) in 5% CO2 in humidified atmosphere at 37 °C. Finally, cells were treated with 0,1% BSA-PBS (control), TNF-α (R&D Systems), IFN-γ (Peprotech) or the mixture.

### Statistical analyses

Unless otherwise specified, two group comparisons were performed by Mann–Whitney’s U-test (nonparametric comparison of two independent series). For primary mixed glial cell cultures experiments, multiple group comparisons were performed by Kruskal-Wallis test followed by a pair wise Dunn’s post hoc test (nonparametric comparison of multiple independent series). A p-value < 0.05 was considered as statistically significant (***p < 0.001, **p < 0.01; *p < 0.05). The values are expressed as the mean ± SEM.

A complementary description of material and methods used in this article is provided in the supplementary material.

### Data availability statement

All data are fully available upon request.

## Electronic supplementary material

Supplementary information

## References

[1] Barouki R, Coumoul X, Fernandez-Salguero PM (2007). The aryl hydrocarbon receptor, more than a xenobiotic-interacting protein. FEBS Lett..

[2] Choudhary, M. *et al*. Aryl hydrocarbon receptor knock-out exacerbates choroidal neovascularization via multiple pathogenic pathways. *J*. *Pathol*., doi:10.1002/path.4433 (2014). 10.1002/path.4433 PMC4277859 25186463

[3] Mulero-Navarro S, Fernandez-Salguero PM (2016). New Trends in Aryl Hydrocarbon Receptor Biology. Front. Cell Dev. Biol..

[4] Abbott BD, Birnbaum LS, Perdew GH (1995). Developmental expression of two members of a new class of transcription factors: I. Expression of aryl hydrocarbon receptor in the C57BL/6N mouse embryo. Dev. Dyn. Off. Publ. Am. Assoc. Anat..

[5] Williamson MA, Gasiewicz TA, Opanashuk LA (2005). Aryl hydrocarbon receptor expression and activity in cerebellar granule neuroblasts: implications for development and dioxin neurotoxicity. Toxicol. Sci. Off. J. Soc. Toxicol..

[6] Latchney SE, Hein AM, O’Banion MK, DiCicco-Bloom E, Opanashuk LA (2013). Deletion or activation of the aryl hydrocarbon receptor alters adult hippocampal neurogenesis and contextual fear memory. J. Neurochem..

[7] Petersen SL (2000). Distribution of mRNAs encoding the arylhydrocarbon receptor, arylhydrocarbon receptor nuclear translocator, and arylhydrocarbon receptor nuclear translocator-2 in the rat brain and brainstem. J. Comp. Neurol..

[8] Filbrandt CR, Wu Z, Zlokovic B, Opanashuk L, Gasiewicz TA (2004). Presence and functional activity of the aryl hydrocarbon receptor in isolated murine cerebral vascular endothelial cells and astrocytes. Neurotoxicology.

[9] Chevallier A (2013). Oculomotor deficits in aryl hydrocarbon receptor null mouse. PloS One.

[10] Richards MD, Wong A (2015). Infantile nystagmus syndrome: clinical characteristics, current theories of pathogenesis, diagnosis, and management. Can. J. Ophthalmol. J. Can. Ophtalmol..

[11] Yonehara K (2016). Congenital Nystagmus Gene FRMD7 Is Necessary for Establishing a Neuronal Circuit Asymmetry for Direction Selectivity. Neuron.

[12] Trobe JD, Sharpe JA, Hirsh DK, Gebarski SS (1991). Nystagmus of Pelizaeus-Merzbacher disease. A magnetic search-coil study. Arch. Neurol..

[13] Hickman SJ, Raoof N, McLean RJ, Gottlob I (2014). Vision and multiple sclerosis. Mult. Scler. Relat. Disord..

[14] Leigh, R. J. & Zee, D. S. The Neurology of Eye Movements. (Oxford University Press, 2015).

[15] Beraneck, M., Lambert, F. M. & Sadeghi, S. G. Functional development of rodent vestibular system: sensorimotor pathways for stabilization of gaze and posture. In R. Romand & I. Varela-Nieto (Eds), Development of Auditory and Vestibular Systems: Academic Press 449–488 (Academic Press, 2014).

[16] Rick JM, Horschke I, Neuhauss SC (2000). Optokinetic behavior is reversed in achiasmatic mutant zebrafish larvae. Curr. Biol. CB.

[17] Huang Y-Y, Rinner O, Hedinger P, Liu S-C, Neuhauss SCF (2006). Oculomotor instabilities in zebrafish mutant belladonna: a behavioral model for congenital nystagmus caused by axonal misrouting. J. Neurosci. Off. J. Soc. Neurosci..

[18] Williams RW, Hogan D, Garraghty PE (1994). Target recognition and visual maps in the thalamus of achiasmatic dogs. Nature.

[19] Long M, Jiang W, Liu D, Yao H (2015). Contrast-dependent orientation discrimination in the mouse. Sci. Rep..

[20] Barton JJ, Cox TA, Digre KB (1999). Acquired convergence-evoked pendular nystagmus in multiple sclerosis. J. Neuro-Ophthalmol. Off. J. North Am. Neuro-Ophthalmol. Soc..

[21] Baumann N, Pham-Dinh D (2001). Biology of oligodendrocyte and myelin in the mammalian central nervous system. Physiol. Rev..

[22] Wang Y, Wu TR, Cai S, Welte T, Chin YE (2000). Stat1 as a component of tumor necrosis factor alpha receptor 1-TRADD signaling complex to inhibit NF-kappaB activation. Mol. Cell. Biol..

[23] Fernández M (2010). A single prenatal exposure to the endocrine disruptor 2,3,7,8-tetrachlorodibenzo-p-dioxin alters developmental myelination and remyelination potential in the rat brain. J. Neurochem..

[24] Mayo L (2014). Regulation of astrocyte activation by glycolipids drives chronic CNS inflammation. Nat. Med..

[25] Quintana FJ (2008). Control of T(reg) and T(H)17 cell differentiation by the aryl hydrocarbon receptor. Nature.

[26] Huai W (2014). Aryl hydrocarbon receptor negatively regulates NLRP3 inflammasome activity by inhibiting NLRP3 transcription. Nat. Commun..

[27] Kimura A (2009). Aryl hydrocarbon receptor in combination with Stat1 regulates LPS-induced inflammatory responses. J. Exp. Med..

[28] Li J, Parker B, Martyn C, Natarajan C, Guo J (2013). The PMP22 gene and its related diseases. Mol. Neurobiol..

[29] Lossos A (2015). Myelin-associated glycoprotein gene mutation causes Pelizaeus-Merzbacher disease-like disorder. Brain J. Neurol..

[30] Fernandez-Salguero P (1995). Immune system impairment and hepatic fibrosis in mice lacking the dioxin-binding Ah receptor. Science.

[31] Launay, P.-S. *et al*. Combined 3DISCO clearing method, retrograde tracer and ultramicroscopy to map corneal neurons in a whole adult mouse trigeminal ganglion. *Exp*. *Eye Res*., doi:10.1016/j.exer.2015.06.008 (2015). 10.1016/j.exer.2015.06.008 26072022

[32] Ertürk A (2012). Three-dimensional imaging of solvent-cleared organs using 3DISCO. Nat. Protoc..

[33] Tomkiewicz C (2013). The aryl hydrocarbon receptor regulates focal adhesion sites through a non-genomic FAK/Src pathway. Oncogene.

[34] Bolstad BM, Irizarry RA, Astrand M, Speed TP (2003). A comparison of normalization methods for high density oligonucleotide array data based on variance and bias. Bioinforma. Oxf. Engl..

[35] Kaye, J. *et al*. Laquinimod arrests experimental autoimmune encephalomyelitis by activating the aryl hydrocarbon receptor. *Proc*. *Natl*. *Acad*. *Sci*. *USA*, doi:10.1073/pnas.1607843113 (2016). 10.1073/pnas.1607843113 PMC5068259 27671624

