# Supplementary material for: AhR-deficiency as a cause of demyelinating disease and inflammation
Source: Sci Rep. 2017 Aug 29;7:9794. doi: 10.1038/s41598-017-09621-3 (PMC5575046; doi:10.1038/s41598-017-09621-3)
Supplement: Supplementary file 1 — Supplementary information [file 41598_2017_9621_MOESM1_ESM.pdf]

Supplementary information

Title: AhR-deficiency as a cause of demyelinating disease and inflammation

Authors: Ludmila Juricek*[,1,6], Julie Carcaud*[,2,6], Alice Pelhaitre[1,6], Thorfinn T. Riday[5,6], Aline Chevallier[1,6], Justine Lanzini[3,6], Nicolas Auzeil[3,6], Olivier Laprévote[3,6], Florent Dumont[4,6], Sebastien Jacques[4,6], Frank Letourneur[4,6], Charbel Massaad[1,6], Cendra Agulhon[5,6], Robert Barouki[1,6], Mathieu Beraneck**[,2,6], Xavier Coumoul**[,1,6]

Table of contents

Page 2 **Supplementary methods**

Pages 5-6 **Table S1**

Pages 7-9 **Table S2**

Page 10 **Table S3**

Page 11 **Legend Figure S1**

Page 12 **Legend Figure S2**

Page 13 **Legend Complete Western Blot**

Supplementary methods

Lipidomic analysis

Chemicals and reagents

Chloroform (CarloErbaReactifs SDS, France), acetonitrile, methanol - MeOH, isopropanol of LC-MS grade (J.T. Baker, Phillipsburg, NJ, USA), 3,5-di-tert-4-butylhydroxytoluene - BHT, ammonium acetate, sodium chloride - NaCl (Sigma-Aldrich, France), methyl tert-butyl ether – MTBE (Acros Organics - Thermo Fisher Scientific, France), were used to prepare ONs extracts, standard lipid solutions and mobile phase for reverse phase liquid chromatography. All standard lipids were obtained from Avanti Polar Lipids, Inc. (Alabaster, AL, USA) and are listed in the tables S2a and S2b.

Sample preparation and lipid extraction

All the organic solvents used for lipid extraction and resuspension content 0.01 % *w/v* 3,5-di-tert-4-butylhydroxytoluene. Weighted ONs were ground in ice cold water with NaCl (350 mg/mL) with a Precellys®24-Dual (Precellys - Bertin Technologies) at 5,000 rpm for 30 s. Each ONs homogenate was extracted with MeOH and MTBE. Following centrifugation (1,000 rpm for 10 min), the organic layer was collected and evaporated to dryness under reduced pressure at 37 °C (SpeedVac, Thermo Fisher Scientific). Lipid extracts were re-suspended in a 35:35:20:10 *v/v/v/v* acetonitrile/isopropanol/chloroform/water solution (190 µL).

Lipidomic analysis of lipid extracts

UPLC-MS analysis: a quality control QC-1/1 was prepared by pooling 25 µL of each re-suspended lipid extract and a fraction was diluted to one-third and one-sixth to give QC-1/3 and QC-1/6, respectively. All samples and QCs were placed in an autosampler at 10 °C and analyzed by reversed phase ultra-performance liquid chromatography (RP-UPLC) coupled to a hybrid quadrupole-orthogonal time-of-flight mass spectrometer equipped with an electrospray ionization (ESI) source (ACQUITY UPLC® and SYNAPT® G2 High Definition MS™ mass spectrometer, Waters, Manchester, UK). This technique will be referred to UPLC-ESI-MS hereafter. Lipids chromatographic separation was performed on a CSH C18 1.7 µm column (2.1 x 100 mm) sed at 50 °C. Lipids were eluted with 4:6 *v/v* acetonitrile/water containing 10 mM ammonium acetate (A) and 1:9 *v/v* acetonitrile/isopropanol containing 10 mM ammonium acetate (B) at a flow rate of 0.40 mL/min. The percentage of phase B increased from 40 % to 100 % (curve 6) over 10 min and was held at 100 % for 2 min before a rapid

return to 40 % followed by an equilibration period of 2.5 min. Data were collected separately both in positive (ESI+) and negative (ESI-) ion modes. ESI source parameters were as follows: source temperature 120 °C, desolvation temperature 450 °C, cone gas flow 20 L/h, desolvation gas flow 800 L/h, capillary voltage for ESI+ ion mode 3,000V, for ESI- ion mode 2,400 V, cone voltage 30 V for ESI+ and 45 V for ESI-. The mass spectrometer was operated in the "MS resolution mode" of acquisition for both polarities. Centroided accurate mass spectra were acquired over the *m/z* range 50-1000 with a scan time of 0.1 s and an interscan delay of 0.01 s using a target mass resolution of 21,500 (FWHM as defined at *m/z* 500). Mass measurements were corrected during acquisition using an external reference (Lock-Spray™) comprising a 2 ng/µL solution of leucine enkephalin in acetonitrile/water 50:50, with an analyte-to-reference scan ratio of 20:1. Undiluted quality control samples were injected at the beginning of the batch to condition the chromatographic column. Samples were randomly analyzed and injected in triplicate. A mixture of 48 standard lipids belonging to 10 of the main lipid classes (fatty acids FAs, phosphatidic acids PAs, phosphatidylethanolamines PEs, phosphatidylserines PSs, phosphatidylcholines PCs, phosphatidylglycerols PGs, ceramides CERs, sphingomyelins SMs, diacylglycerols DGs and triacylglycerols TGs), at a final individual concentration of 1 µM, was also periodically injected throughout the analytical batch. The above mixture of lipids was used to create an in-house database compiling the retention time (t_R) values against equivalent number of carbon (ECN) for each main class and sub-class of lipids.

Data pre-processing: raw data files acquired on the UPLC-MS platform were converted to .mzData format using the MassWolf script under R. XCMS set up with parameters suitable for high resolution LC-MS data sets in centroid mode was used for feature detection, feature matching and retention time alignment across all mass spectra files. Subsequent to data pre-processing, a matrix listing peak areas associated to a unique *m/z* and retention time *vs* samples and QC, was generated. An ONs weight normalization followed by a total intensity normalization in both ionization modes were performed. The table was then filtered by selecting only variables with a coefficient of variation (CV) inferior to 30 % in the QC-1/1 and a correlation to the dilution (r) superior to 0.7 in the QC-1/1, QC-1/3 and QC-1/6. The final table was then exported for statistical analysis.

Univariate data analysis: A comparison between AhR KO and WT were assessed with the Wilcoxon (Mann-Whitney) test. The p-value were adjusted for multiple comparison by controlling the false discovery rate at a 5 % threshold.

Multivariate data analysis: Unsupervised and supervised multivariate analyses were performed using SIMCA-P+ software version 13.0.3 (Umetrics, Umeå, Sweden). A pareto scaling was applied to the variables prior to unsupervised principal component analysis (PCA) and supervised partial least squares-discriminant analysis (PLS-DA). A permutation test with 999 times permutations on the class labels was conducting to measure over-fitting of the model. Moreover, an orthogonal partial least squares-discriminant analysis (OPLS-DA) model was built based on the PLS-DA model. An S-plot was created from the OPLS-DA model to investigate the lipids related to the statistically significant differences between AhR KO and WT mice. A cross-validated analysis of variance (CV-ANOVA) was carried out on each supervised model to assess the statistical significance of group separation. Moreover, R2 and Q2 quality metrics superior to 0.4 traduced an acceptable biological model. Only variables with a VIP>1 and a p(corr)>|0.6| were considered significant.

Lipid structure assignment: The structure assignment of lipids was based on their m/z and their retention time. LIPID MAPS and METLIN data bases were requested using the mass accuracy with a tolerance window of 5 ppm. A linear relationship between retention time and ECN for each lipid class was determined allowing to determine a theoretical retention time for each lipid. A relative difference of the retention time below 15 % was considered acceptable to confirm the lipid annotation.

Lipid annotation	p-value	AhR KO/WT (%)	Up-regulated (UR) or Down-regulated (DR) in AhR-KO mice	Adduct detected
DG(36:1)	**	24	DR	$[M+Na]^+$
TG(52:6)	**	27	DR	$[M+NH_4]^+$
GalCer(d36:1)	***	22	DR	$[M+H]^+$, $[M-H]^-$
GalCer(d41:2)	**	24	DR	$[M+CH_3COO]^-$
GalCer(d42:1)	*	16	DR	$[M-H]^-$
GalCer(d42:2)	**	22	DR	$[M-H]^-$, $[M+H]^+$,$[M+CH_3COO]^-$
PC(30:0)	**	23	DR	$[M+H]^+$
PC(34:0)	*	27	UR	$[M+K]^+$
PC(36:1)	**	12	DR	$[M+H]^+$
PC(38:4)	***	33	UR	$[M+Na]^+$
PC(P-40:0)/PC(O-40:1)	**	29	UR	$[M+K]^+$
PC(P-42:3)/PC(O-42:4)	*	7	DR	$[M+K]^+$
PE(34:0)	***	26	DR	$[M+H-H_2O]^+$, $[M-H_2O-H]^-$
PE(34:1)	*	12	DR	$[M+H]^+$
PE(36:0)	**	33	DR	$[M-H_2O-H]^-$
PE(36:1)	**	16	DR	$[M+H-H_2O]^+$, $[M+H]^+$,$[M+Na]^+$,$[M-H_2O-H]^-$,$[M-H]^-$
PE(36:2)	**	41	DR	$[M+H-H_2O]^+$, $[M+H]^+$,$[M-H]^-$
PE(38:0)	**	18	DR	$[M+H-H_2O]^+$, $[M-H_2O-H]^-$
PE(38:1)	*	12	DR	$[M+CH_3COO]^-$
PE(38:2)	*	20	DR	$[M+H]^+$
PE(38:3)	**	18	DR	$[M+H-H_2O]^+$
PE(38:4)	**	25	UR	$[M+Na]^+$

PE(40:3)	**	15	DR	$[M+H-H_2O]^+$
PE(40:4)	***	50	UR	$[M+Na]^+$, $[M+H]^+$,$[M+H-H_2O]^+$,$[M-H]^-$
PE(P-38:4)/PE(O-38:5)	**	24	DR	$[M+CH_3COO]^-$
PE(P-40:4)/PE(O-40:5)	**	14	DR	$[M+CH_3COO]^-$
PE-Cer(d40:2)	**	34	DR	$[M+Na]^+$
PS(36:1)	*	24	DR	$[M-H]^-$, $[M+H]^+$

Table S1: Lipid composition of the myelin sheath in WT and AhR-KO mice. DG: diacylglycerol; GalCer: galactosyl-ceramide; PC: phosphatidylcholine; PE: phosphatidylethanolamine; PE-Cer: phosphatidylethanolamine-ceramide; PS: phosphatidylserine; TG: triacylglycerol. Univariate analyses was performed and the p-values were adjusted for multiple comparison by controlling the false discovery rate at a 5 % threshold (*** p-value<0.001, ** p-value<0.01; * p-value<0.05). Multivariate statistical analyses were also performed. The OPLS-DA models were validated with, for the negative ion mode: R2X = 0.71, R2Y = 0.89, Q2 = 0.77 and p-value (CV-ANOVA) = 10-4, and for the positive ion mode: R2X = 0.68, R2Y = 0.98, Q2 = 0.86 and p-value (CV-ANOVA) = 10-5. Each discriminant variables had a VIP>1 and a p(corr)>|0.6|.

Table S2: Lipid composition of the standard mix (individual concentration 1 µM). a. first part**. b.** second part.

Table S2a: Lipid composition of standard mix (individual concentration 1 µM): part 1

Lipids category	Name	Molecular formula
Free fatty acids		
FA(16:0)	palmitic acid	$C_{16}H_{32}O_2$
FA(18:0)	stearic acid	$C_{18}H_{36}O_2$
Ceramides		
Cer (d18:1/8:0)	N-(octanoyl)-sphing-4-enine	$C_{26}H_{51}NO_3$
Cer(d18:1/14:0)	N-(tetradecanoyl)-sphing-4-enine	$C_{32}H_{63}NO_3$
Cer(d18:1/16:0)	N-(hexadecanoyl)-sphing-4-enine	$C_{34}H_{67}NO_3$
Cer(d18:1/17:0)	N-(heptadecanoyl)-sphing-4-enine	$C_{35}H_{69}NO_3$
Cer(d18:1/18:0)	N-(octadecanoyl)-sphing-4-enine	$C_{36}H_{71}NO_3$
CerP(d18:1/16:0)	N-(hexadecanoyl)-sphing-4-enine-1-phosphate	$C_{34}H_{68}NO_6P$
Sphyngomyelins		
SM(d18:1/16:0)	N-(hexadecanoyl)-sphing-4-enine-1-phosphocholine	$C_{39}H_{79}N_2O_6P$
SM(d18:1/24:1)	N-(tetracosenoyl)-sphing-4-enine-1-phosphocholine	$C_{47}H_{93}N_2O_6P$
Diacylglycerols		
DG(18:1/18:1/0:0)	1-2-dioleoyl-*sn*-glycerol	$C_{39}H_{72}O_5$
DG(16:0/18:1/0:0)	1-palmitoyl-2-oleoyl-*sn*-glycerol	$C_{37}H_{70}O_5$
DG(14:0/14:0/0:0)	1,2-ditetradecanoyl-*sn*-glycerol	$C_{31}H_{60}O_5$
Triacylglycerols		
TG(18:2/18:2/18:2)	1,2,3-trioctadecadienoyl-glycerol	$C_{57}H_{98}O_6$

Table S2b: Lipid composition of standard mix (individual concentration 1 µM): part 2

Lipids category	Name	Molecular formula
Phosphatidic acids		
PA(14:0/14:0)	1,2-ditetradecanoyl-*sn*-glycero-3-phosphate	$C_{31}H_{61}O_8P$
PA(16:0/16:0)	1,2-dihexadecanoyl-*sn*-glycero-3-phosphate	$C_{35}H_{69}O_8P$
PA(18:0/18:0)	1,2-dioctadecanoyl-*sn*-glycero-3-phosphate	$C_{39}H_{75}O_8P$
PA(16:0/18:1)	1-palmitoyl-2-oleoyl-sn-glycero-3-phosphate	$C_{37}H_{71}O_8P$
PA(17:0/17:0)	1,2-diheptadecanoyl-*sn*-glycero-3-phosphate	$C_{37}H_{73}O_8P$
PA(16:0/0:0)	1-hexadecanoyl-sn-glycero-3- phosphate	$C_{19}H_{39}O_7P$
Phosphatidylcholines		
PC(17:0/17:0)	1,2-diheptadecanoyl-*sn*-glycero-3-phosphocholine	$C_{42}H_{84}NO_8P$
PC(18:0/18:2)	1-stearoyl-2-linoleoyl-*sn*-glycero-3-phosphocholine	$C_{44}H_{84}NO_8P$
PC(18:1/18:1)	1,2-dioleoyl-sn-glycero-3-phosphocholine	$C_{44}H_{84}NO_8P$
PC(20:1/20:1)	1,2-dieicosenoyl-*sn*-glycero-3-phosphocholine	$C_{48}H_{92}NO_8P$
PC(O-18:0/O-18:0)	1,2-di-O-octadecyl-*sn*-glycero-3-phosphocholine	$C_{44}H_{92}NO_6P$
PC(16:0/0:0)	1-hexadecanoyl-sn-glycero-3-phosphocholine	$C_{24}H_{50}NO_7P$
Phosphatidylethanolamines		
PE(14:0/14:0)	1,2-ditetradecanoyl-*sn*-glycero-3-phosphoethanolamine	$C_{33}H_{66}NO_8P$
PE(16:0/16:0)	1,2-dipalmitoyl-sn-glycero-3-phosphoethanolamine	$C_{37}H_{74}NO_8P$
PE(18:0/18:0)	1,2-distearoyl-sn-glycero-3-phosphoethanolamine	$C_{41}H_{82}NO_8P$
PE(16:1/16:1)	1,2-dipalmitoleoyl-*sn*-glycero-3-phosphoethanolamine	$C_{37}H_{70}NO_8P$
PE(18:0/18:1)	1-stearoyl-2-oleoyl-*sn*-glycero-3-phosphoethanolamine	$C_{41}H_{80}NO_8P$
PE(17:0/17:0)	1,2-diheptadecanoyl-*sn*-glycero-3-phosphoethanolamine	$C_{39}H_{78}NO_8P$
PE(14:0/0:0)	1-tetradecanoyl-sn-glycero-3-phosphoethanolamine	$C_{19}H_{40}NO_7P$
PE(16:0/0:0)	1-hexadecanoyl-sn-glycero-3-phosphoethanolamine	$C_{21}H_{44}NO_7P$
PE(18:0/0:0)	1-octadecanoyl-*sn*-glycero-3-phosphoethanolamine	$C_{23}H_{48}NO_7P$
Phosphatidylglycerols		

PG(14:0/14:0)	1,2-ditetradecanoyl-*sn*-glycero-3-phospho-(1'-*rac*-glycerol)	$C_{34}H_{67}O_{10}P$
PG(16:0/16:0)	1,2-dipalmitoyl-*sn*-glycero-3-phospho-(1'-*rac*-glycerol)	$C_{38}H_{75}O_{10}P$
PG(18:0/18:0)	1,2-distearoyl-sn-glycero-3-phospho-(1'-rac-glycerol)	$C_{42}H_{83}O_{10}P$
PG(18:2/18:2)	1,2-dilinoleoyl-*sn*-glycero-3-phospho-(1'-*rac*-glycerol)	$C_{42}H_{75}O_{10}P$
PG(17:0/17:0)	1,2-diheptadecanoyl-*sn*-glycero-3-phospho-(1'-*rac*-glycerol)	$C_{40}H_{79}O_{10}P$
PG(16:0/18:1)	1-palmitoyl-2-oleoyl-sn-glycero-3-phospho-(1'-rac-glycerol)	$C_{40}H_{77}O_{10}P$
PG(16:0/0:0)	1-hexadecanoyl-sn-glycero-3- phospho-(1'-rac-glycerol)	$C_{22}H_{45}O_{9}P$
Phosphatidylserines		
PS(14:0/14:0)	1,2-ditetradecanoyl-*sn*-glycero-3-phospho-L-serine	$C_{34}H_{66}NO_{10}P$
PS(16:0/16:0)	1,2-dipalmitoyl-sn-glycero-3-phospho-L-serine	$C_{38}H_{74}NO_{10}P$
PS(16:0/18:2)	1-palmitoyl-2-linoleoyl-*sn*-glycero-3-phospho-L-serine	$C_{40}H_{74}NO_{10}P$
PS(17:0/17:0)	1,2-diheptadecanoyl-*sn*-glycero-3-phospho-L-serine	$C_{40}H_{78}NO_{10}P$
PS(18:0/0:0)	1-octadecanoyl-sn-glycero-3-phospho-L-serine	$C_{24}H_{48}NO_{9}P$

Table S3: List of primers used for RT-qPCR

Gene	Forward primer 5'>3'	Reverse primer 5'>3'
Mag	ACTGGTGTGTGGCTGAGAAC	GGATTATGGGGGCAAACTC
Mbp	GGGAAGGGAGGACAACAC	CCGATGGAGTCAAGGATG
Plp	AGCAAAGTCAGCCGCAAAACA	CCAGGGAAGCAAAGGGGG
IL1-β	GCCACCTTTTGACAGTGATG	TCTCCACAGCCACAATGAG
IL6	AGACAAAGCCAGAGTCCTTCA	GCCACTCCTTCTGTGACTCC
Mcp-1	AGCAGCAGGTGTCCCAAAGA	ACGGGTCAACTTCACATTCAA
Tnf-α	CCACCACGCTCTTCTGTCTACTGAACTT	GTGGGCTACAGGCTTGTCACTCG
Rantes	AGCAGCAAGTGCTCCAATCT	TACTGAGTGGCATCCCCAAG
Mcp-3	TTCTGTGCCTGCTGCTCATA	TTGACATAGCAGCATGTGGAT
Gapdh	GTGGACCTCATGGCCTACAT	TGTGAGGGAGATGCTCAGTG

SFigure 1

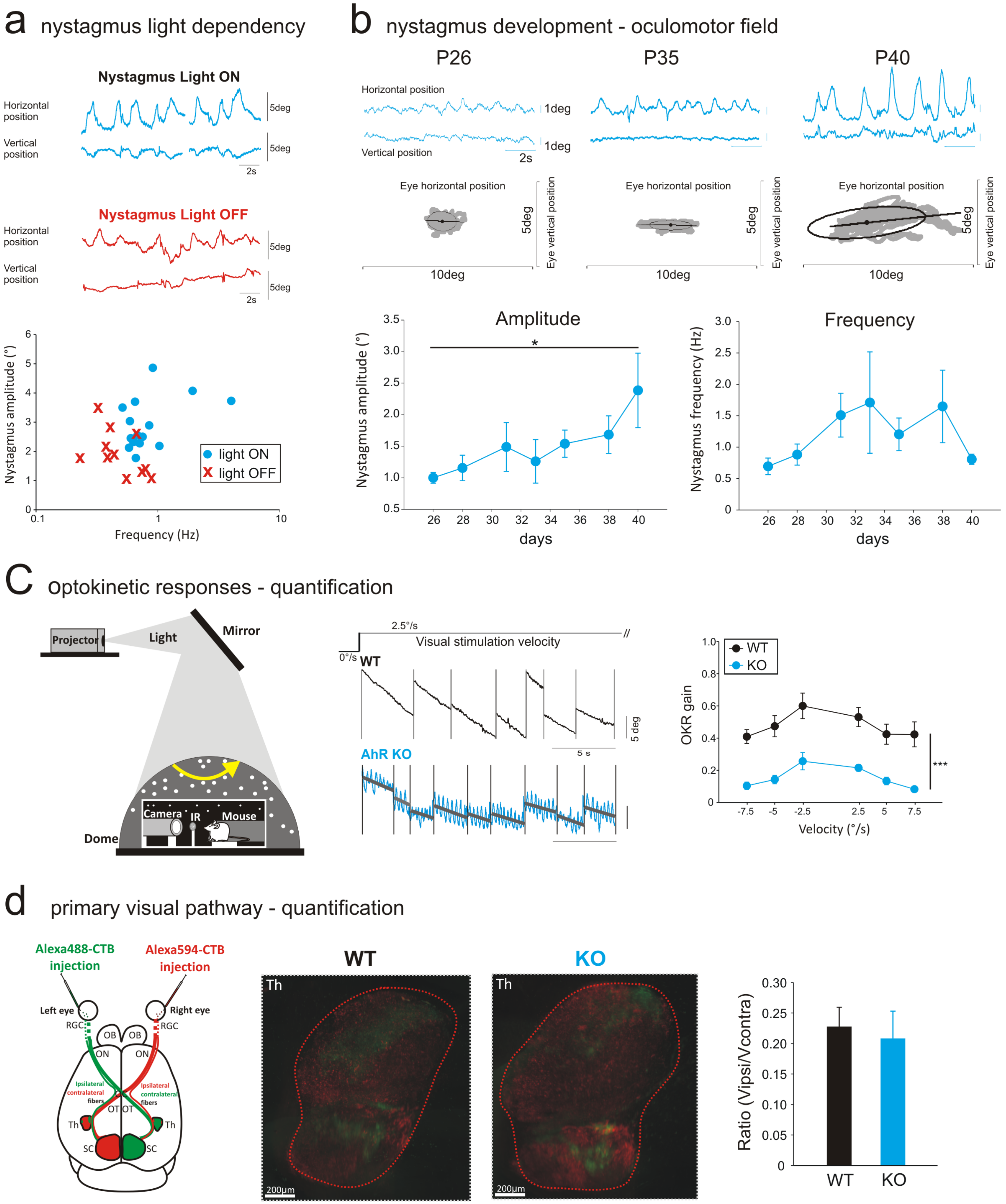
a nystagmus light dependency
Nystagmus Light ON
Horizontal position
Vertical position
5deg
5deg
2s
Nystagmus Light OFF
Horizontal position
Vertical position
5deg
5deg
2s
Nystagmus amplitude (°)
Frequency (Hz)
light ON
light OFF
b nystagmus development - oculomotor field
P26
P35
P40
Horizontal position
Vertical position
1deg
1deg
2s
Eye horizontal position
Eye vertical position
5deg
10deg
Amplitude
Nystagmus amplitude (°)
days
Frequency
Nystagmus frequency (Hz)
days
C optokinetic responses - quantification
Projector
Light
Mirror
Camera
IR
Mouse
Dome
2.5°/s
Visual stimulation velocity
0°/s
WT
AhR KO
5 deg
5 s
WT
KO
OKR gain
Velocity (°/s)
***
d primary visual pathway - quantification
Alexa488-CTB injection
Alexa594-CTB injection
Left eye
Right eye
RGC
OB
ON
Ipsilateral contralateral fibers
OT
Th
SC
WT
KO
Th
200µm
Ratio (Vipsi/Vcontra)
WT
KO

Figure S1: ***Anatomical and functional characterization of AHR-KO visuo-motor deficits*** **(A). Top**: Raw traces of spontaneous eye movements recorded light ON or light OFF (8 week-old AhR KO). **Bottom**: Nystagmus amplitude *vs* nystagmus frequency recorded in the presence of light (circles) and in the dark (cross) (n=12 AhR KO). **(B)**. **Top**: raw traces of spontaneous eye movements of AhR KO mice at different ages (P26, P35 and P40). Representation of the oculomotor fields. Grey points are the combined horizontal and vertical eye positions during the recordings presented on the top. The ellipses represent 95% of the horizontal and vertical eye positions. The inclination of the ellipse was computed as the slope of the linear regression between vertical and horizontal eye positions. **Bottom**: Representation of the nystagmus amplitude (**left**) or frequency (**right**) as a function of age. **(C)**. **Left**: Experimental set-up for the optokinetic measurements. The mouse is head-fixed in a plastic tube in front of a camera and an infrared light (IR), under a translucid dome. The optical stimulation (white dots on black background which moves in a clockwise or a counter clockwise direction) is projected on a mirror, and then oriented on the dome. **Middle**: Examples of raw traces recorded during optokinetic stimulation at 2.5°/s in WT (black line) and AhR KO mice (blue line). **Right**: OKR gain recorded in WT (black circles, n=6) and AhR KO mice (blue circles, n=18) at different stimulation velocities. The OKR gain is significantly decreased in AhR KO mice in comparison to WT mice. **(D). Left**: Scheme of the protocol used for anatomical staining of visual pathways. Dye is injected into the vitreous humor of either the left (Alexa488 coupled to CTB) or the right (Alexa 594 coupled to CTB) eye, to stain the primary relays of ipsilateral and contralateral retinal ganglion cells (RGC): Optic Nerve (ON), Optic Chiasm (OC), Optic Tract (OT), Thalamus (Th), Superior Colliculus (SC) and Olfactory Bulb (OB). **Middle**: Example of left thalamus staining of a WT mouse and an AhR KO mouse. In both WT and AhR KO mice, thalamus staining show a few ipsilateral fibers (green) and a majority of contralateral fibers (red) in both the WT and AhR KO examples. **Right**: Ratio of ipsilateral/contralateral staining volume measured in WT (black, n=5) and AhR KO mice (blue, n=7) (*** $p<0.001$; * $p<0.05$).

SFigure 2

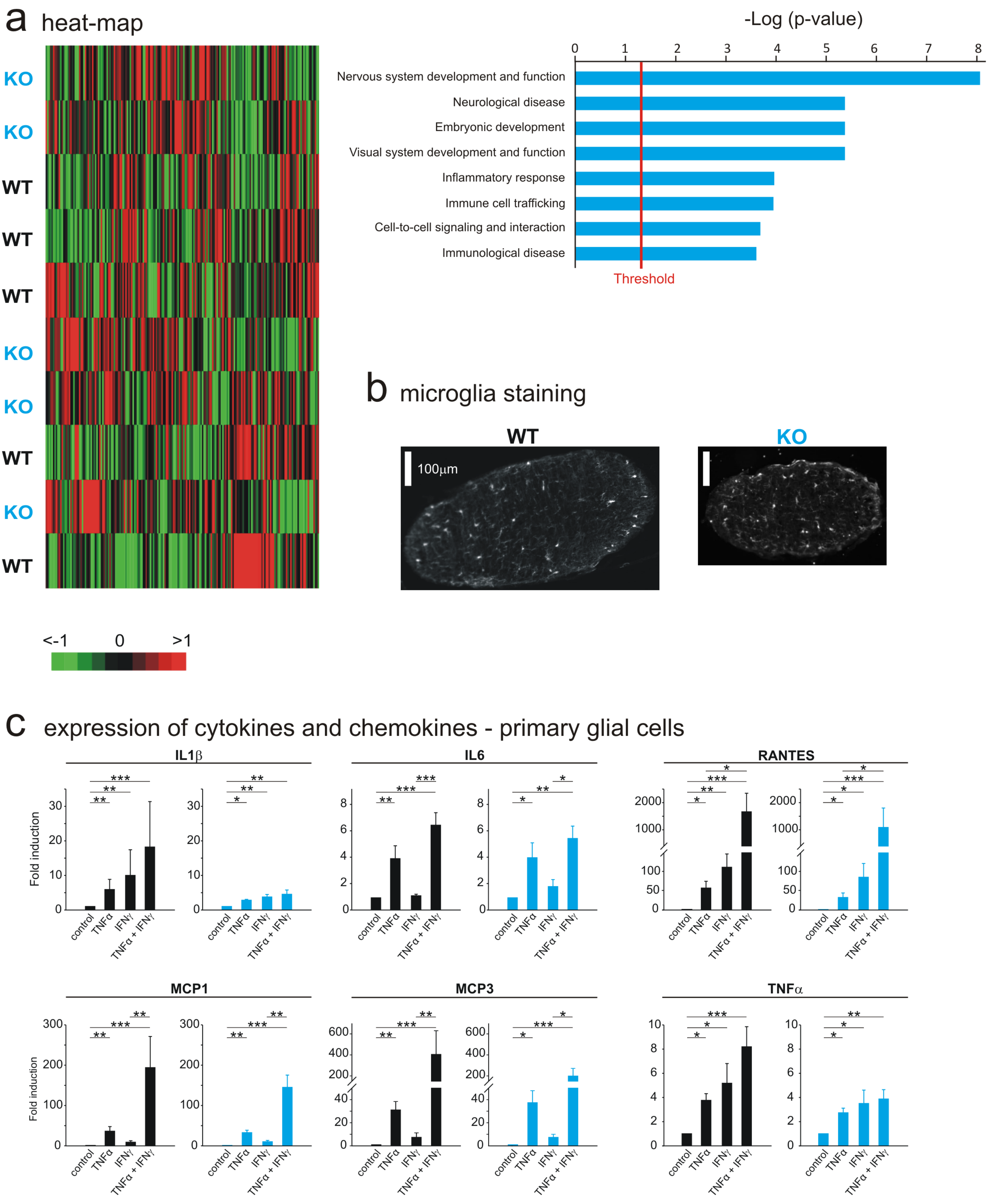
a heat-map
KO
KO
WT
WT
WT
KO
KO
WT
KO
WT
<-1
0
>1
-Log (p-value)
Nervous system development and function
Neurological disease
Embryonic development
Visual system development and function
Inflammatory response
Immune cell trafficking
Cell-to-cell signaling and interaction
Immunological disease
Threshold
b microglia staining
WT
KO
100µm
c expression of cytokines and chemokines - primary glial cells
IL1β
IL6
RANTES
MCP1
MCP3
TNFα
Fold induction
control
TNFα
IFNγ
TNFα + IFNγ

Figure S2: ***Inflammation in AhR-KO mice and potentiation of inflammatory responses in primary mixed glial cells by pro-inflammatory cytokines*** **(A). Left**: Heat map (25.000 genes) diagram showing the expression levels of the differentially expressed genes from the comparison between WT and AhR KO mice optic nerves. **Right**: Gene ontology (GO) analysis of annotated differentially expressed genes between WT and AhR KO mice. Bar charts represent the categories of enriched GO at 8 weeks-old. The threshold indicates the significance of the GO term enrichment in the list of differentially expressed genes (-log (p-value)> 1.3 or p≤ 0.05 is considered significant). **(B)** Expression of immunoreactive IBA1, a microglia marker, in the optic nerve of WT (left) and AhR KO (right) adult mice (Scale bar: 100µm, n=4/group). **(C)** Inflammation genes mRNA expression was determined by Quantitative Real-Time PCR analysis in primary glial cell cultures after 3 days-treatment with TNFα (10ng/mL), IFNγ (10ng/mL), or both (concentration 10ng/mL of each) or vehicle (control, ctrl). The relative mRNA expression levels were represented as relative fold change compared to mRNA abundance in cells treated with vehicle. Data are expressed as the mean of four to six independent experiments. Each experiment consists of pool of 2-8 pups (*** $p<0.001$; ** $p<0.01$; * $p<0.05$).

Complete western blot (cf fig.2)

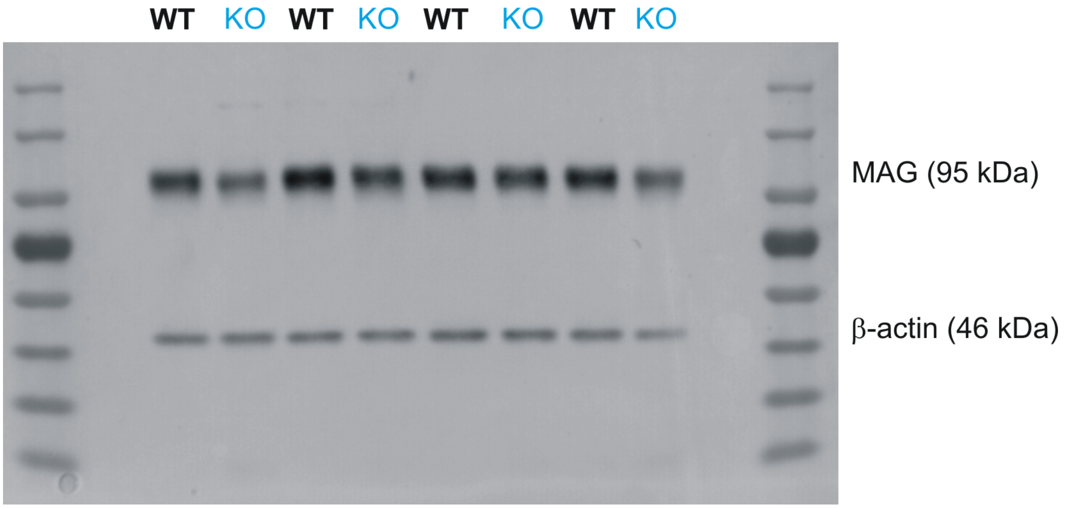

Complete western blot:

MAG protein (95 kDa) in whole tissue lysates of WT and AhR-KO mice optic nerves detected by Western Blot in 4 different experiments. Total actin (46 kDa) was used as a loading control (n=4/group).
